# Supplementary material for: Engineering a membrane protein chaperone to ameliorate the proteotoxicity of mutant huntingtin
Source: Nat Commun. 2025 Jan 17;16:737. doi: 10.1038/s41467-025-56030-6 (PMC11742450; doi:10.1038/s41467-025-56030-6)
Supplement: Supplementary file 1 — Supplementary Information [file 41467_2025_56030_MOESM1_ESM.pdf]

Supplementary Information

Engineering a membrane protein chaperone to ameliorate the proteotoxicity of mutant huntingtin

Jeonghyun Oh[1,#], Christy Catherine[1,#], Eun Seon Kim[2,#], Kwang Wook Min[1,#], Hae Chan Jeong[2], Hyojin Kim[1], Mijin Kim[1], Seung Hae Ahn[1], Nataliia Lukianenko[3], Min Gu Jo[2], Hyeon Seok Bak[1], Sungsu Lim[3], Yun Kyung Kim[3], Ho Min Kim[1,4,*], Sung Bae Lee[2,*], Hyunju Cho[1*]

[1]Center for Biomolecular and Cellular Structure, Institute for Basic Science (IBS), Daejeon 34126, Republic of Korea

[2]Department of Brain Sciences, Daegu Gyeongbuk Institute of Science and Technology (DGIST), Daegu 42988, Republic of Korea

[3]Center for Brain Disorders, Brain Science Institute, Korea Institute of Science and Technology (KIST), Seoul 02792, Republic of Korea

[4]Department of Biological Sciences, Korea Advanced Institute of Science and Technology (KAIST), Daejeon 34141, Republic of Korea

This file includes Supplementary Figures 1-15 and Supplementary Table 1.

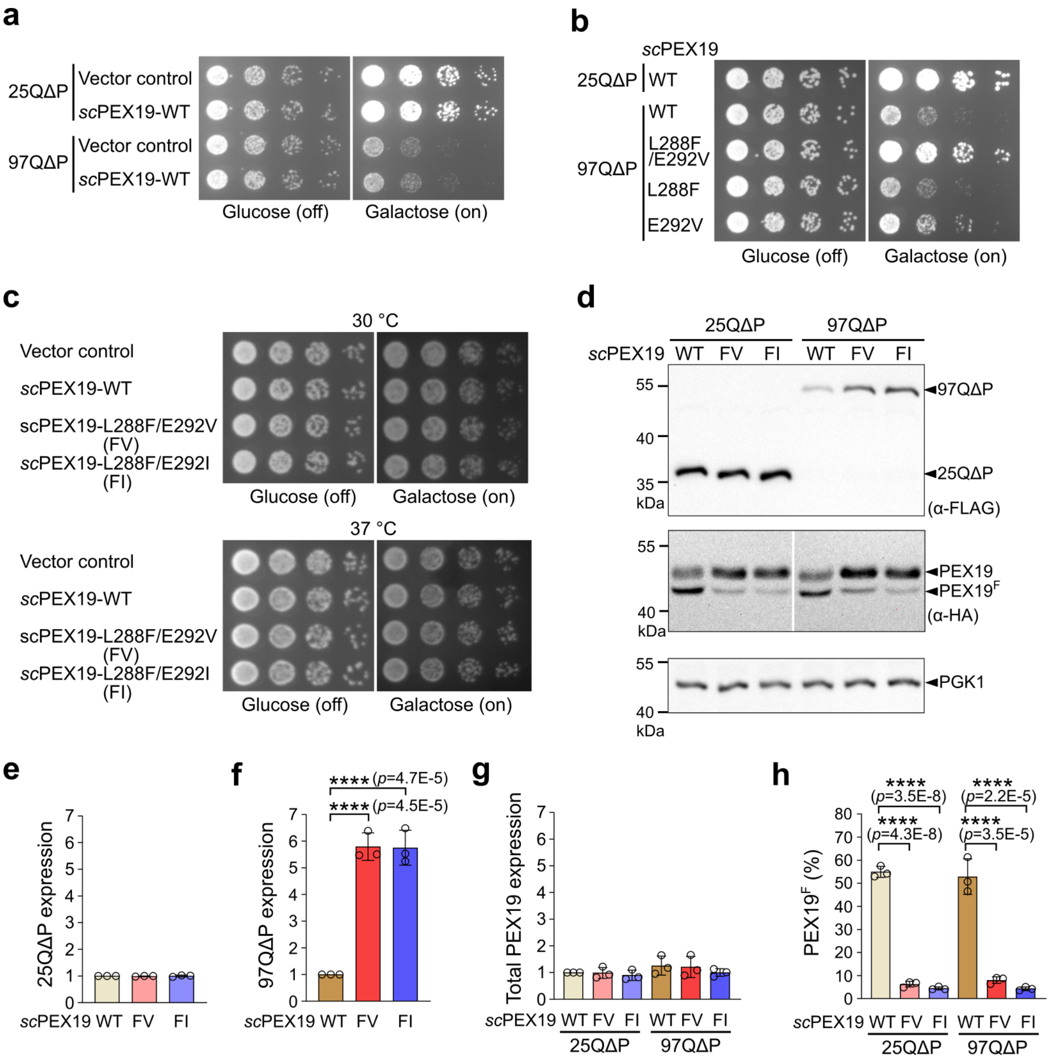

Supplementary Fig. 1. A *sc*PEX19 double mutation (L288F/E292V) is necessary to prevent the cellular toxicity of Httex1-97QΔP-GFP in yeast.

a, **b**, Growth of Httex1-25QΔP-GFP- and Httex1-97QΔP-GFP-integrated yeast cells expressing vector control, *sc*PEX19-WT, *sc*PEX19-FV (L288F/E292V), *sc*PEX19-L288F or *sc*PEX19-E292V. An overnight culture of cells in the selective media containing 2% raffinose and 0.1 % glucose was diluted to an OD_{600} of 0.1. The 5-fold serial diluted cells were spotted onto plates containing galactose or glucose. Representative images from three biological replicates are shown (n=3).

c, Growth test of *sc*PEX19-expressing yeast cells. Vector control, *sc*PEX19-WT, *sc*PEX19-L288F(FV), and *sc*PEX19-L288I(FI)-transformed cells were cultured and spotted as described above. Yeast strains were then incubated at 30°C (normal temperature) or 37°C (mild heat stress). Representative images from three biological replicates are shown (n=3).

d-**h**, Representative images of western blot analysis monitoring the expression levels of Httex1-25QΔP-GFP, Httex1-97QΔP-GFP, *sc*PEX19, and PGK1 are shown in (**d**). Total yeast cell extracts were prepared using the NaOH-SDS lysis method as described in Methods. The protein expression levels of N-terminally Flag-tagged Httex1-25QΔP-GFP and Httex1-97QΔP-GFP were quantified and are shown in (**e**) and (**f**), respectively. (**g**) Total expression levels of HA-tagged *sc*PEX19 were calculated as the intensity sum of both PEX19 and $PEX19^F$ bands and normalized to the sample of Httex1-25QΔP-GFP/*sc*PEX19-WT. (**h**) Relative expression levels of the farnesylated PEX19 (denoted $PEX19^F$) were calculated as (Intensity of $PEX19^F$ band/Total intensity of PEX19 and $PEX19^F$ bands)*100.

All data in (**e**-**h**) are shown as mean ± SD, with three biological replicates (n=3). Pairwise comparisons are shown as indicated, where ****$p < 0.0001$ by the ordinary one-way ANOVA with Tukey post-hoc test.

Source data are provided as a Source Data file.

a ***sc*PEX19-m1**

Forward

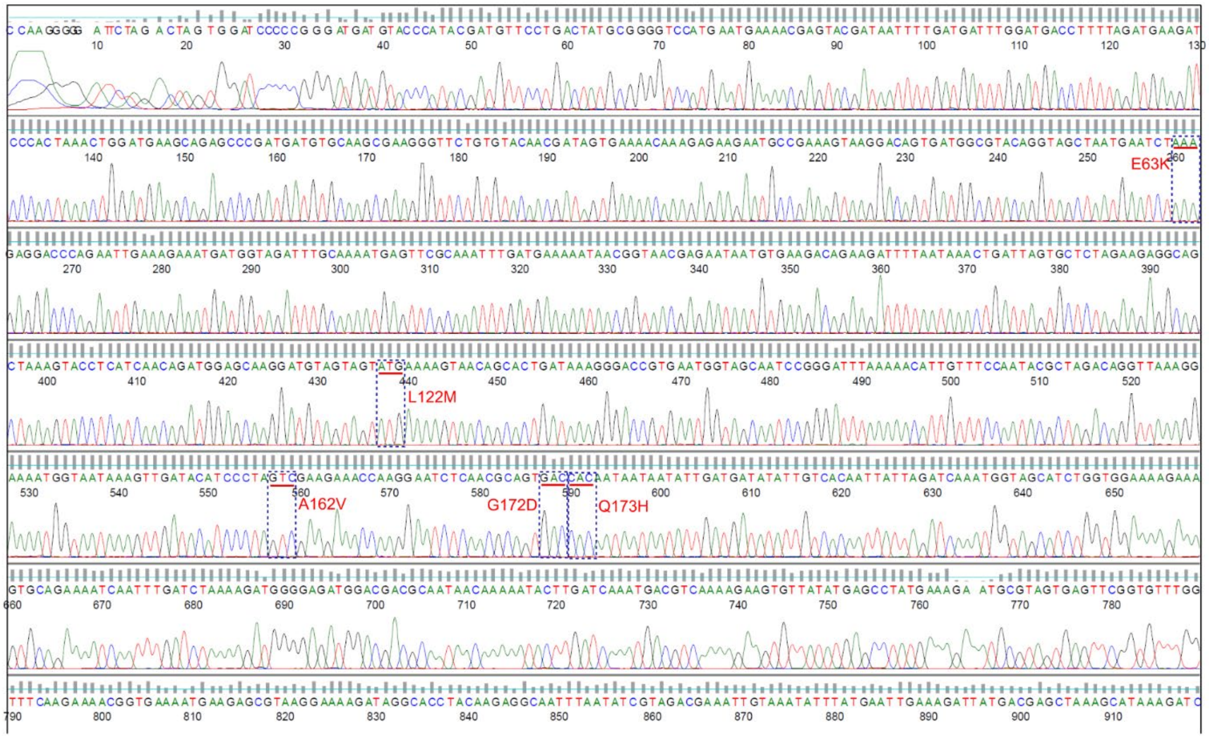

Reverse

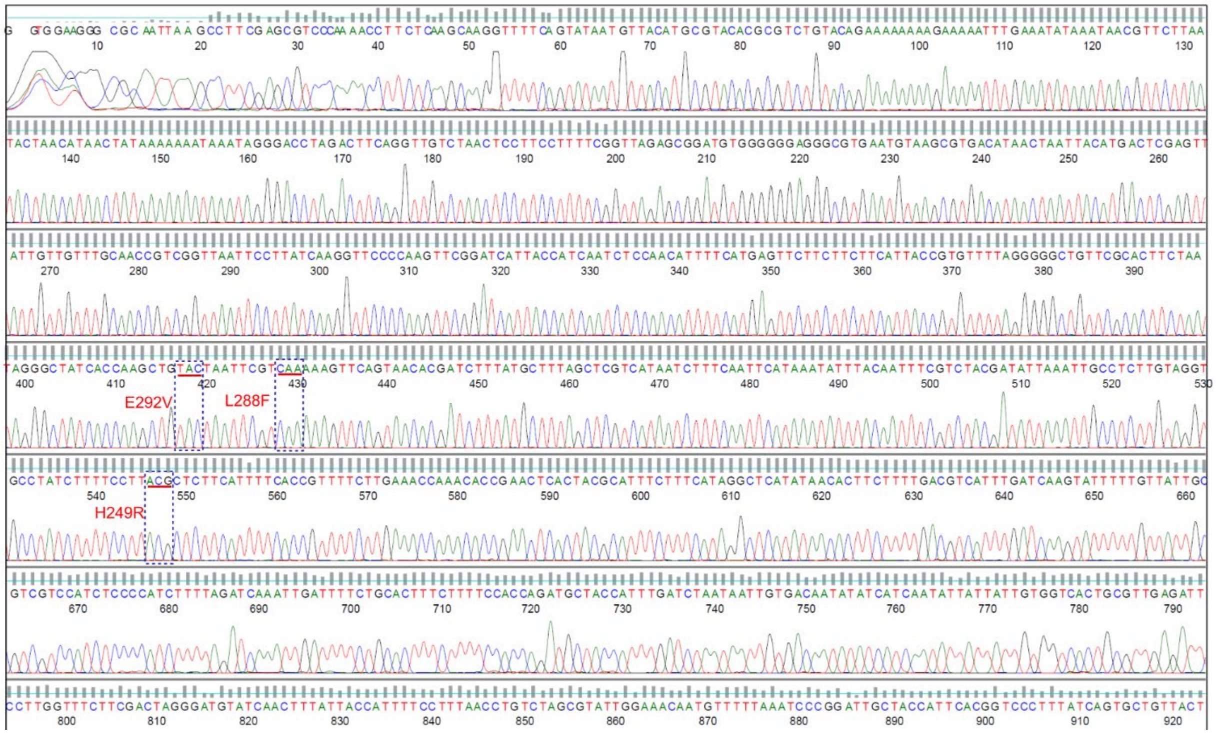

b ***sc*PEX19-m2**

Forward

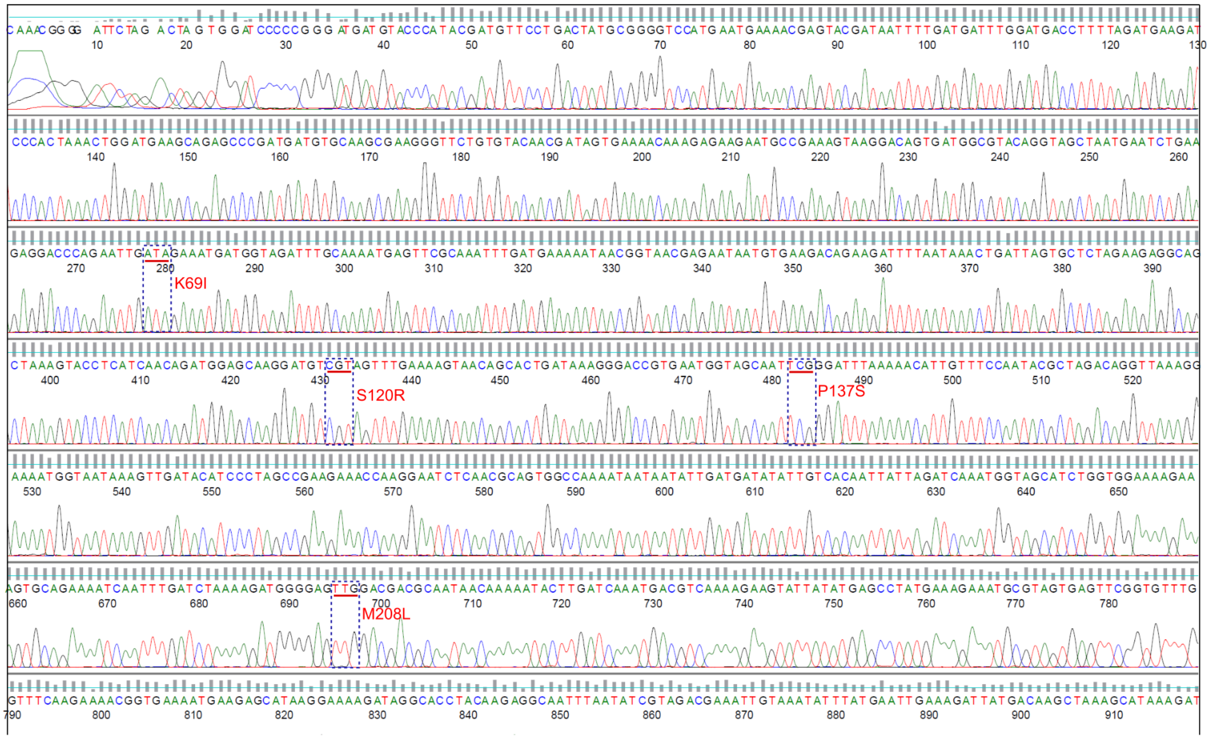

Reverse

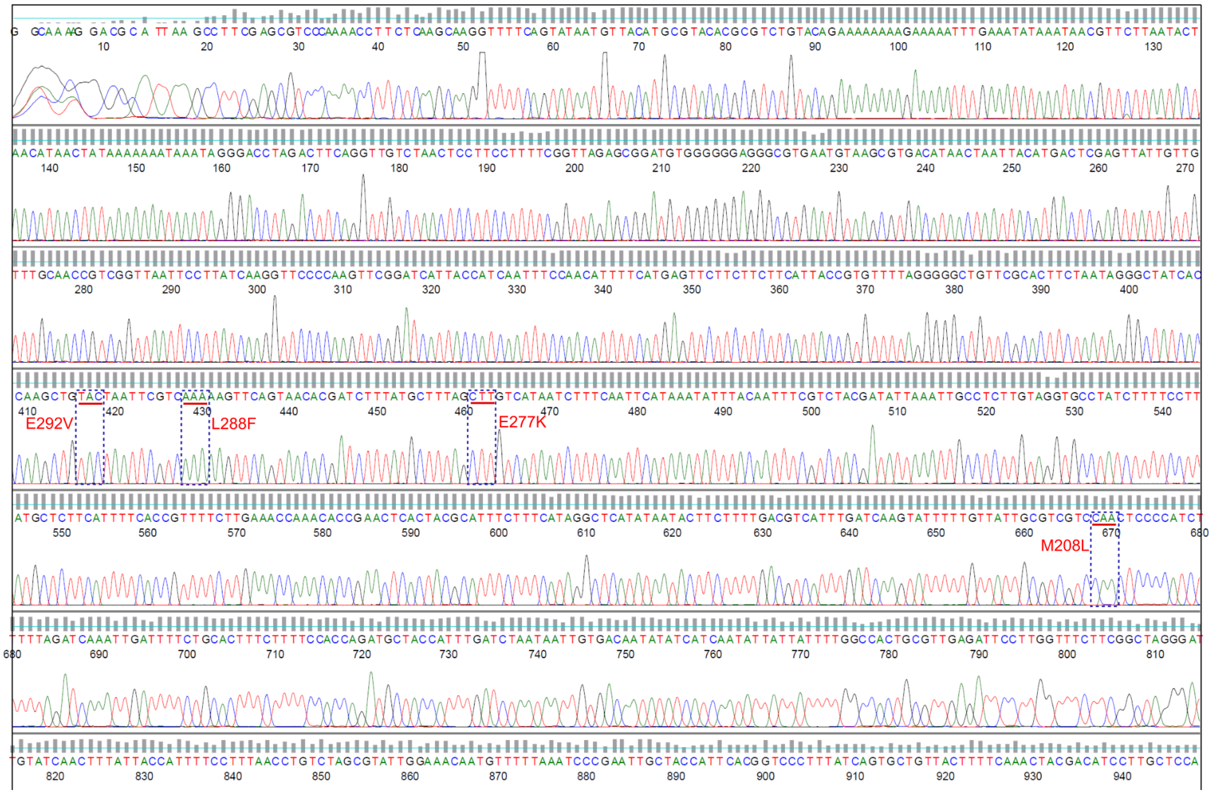

Supplementary Fig. 2. DNA sequencing analysis of the isolated *sc*PEX19-m1 and m2 colonies.

a, **b**, DNA sequence chromatograms of *sc*PEX19-m1 (**a**) and *sc*PEX19-m2 (**b**). Mutation sites in *sc*PEX19-m1 and *sc*PEX19-m1 were highlighted as dotted boxes. All chromatograms for those mutations show single peaks, suggesting that the isolated colonies contain a single mutated *sc*PEX19 gene.

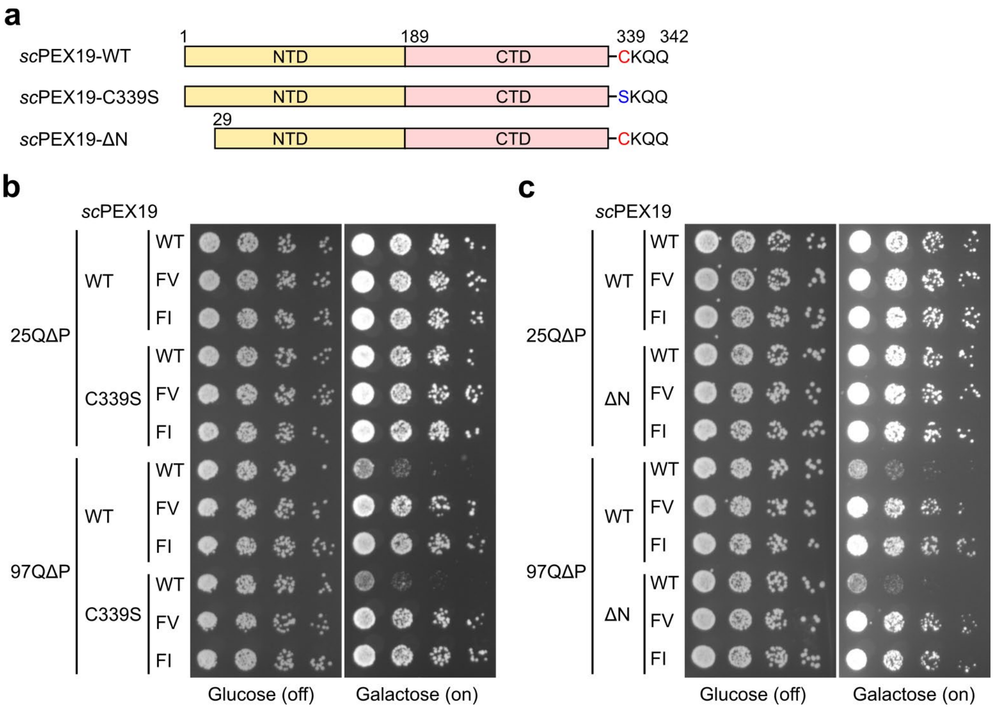

Supplementary Fig. 3. PEX3 binding and farnesylation on *sc*PEX19 variants are dispensable for suppressing the cellular toxicity of Httex1-97QΔP-GFP in yeast.

a, Schematic of the domain organization of *sc*PEX19-WT and its mutants. *sc*PEX19-C339S removes the C-terminal farnesylation site, while *sc*PEX19-ΔN (29-342 aa) lacks residues needed for PEX3 interaction. Either C339S or ΔN mutations were introduced into *sc*PEX19-WT, *sc*PEX19-FV, and *sc*PEX19-FI.

b, **c**, Growth test of Httex1-25QΔP-GFP or Httex1-97QΔP-GFP cells expressing the *sc*PEX19-WT and indicated *sc*PEX19 mutants. Addition of the above mutations to *sc*PEX19-FV and *sc*PEX19-FI did not alter their ability to rescue the yeast cell death caused by Httex1-97QΔP-GFP expression. Representative images from three biological replicates are shown (n=3).

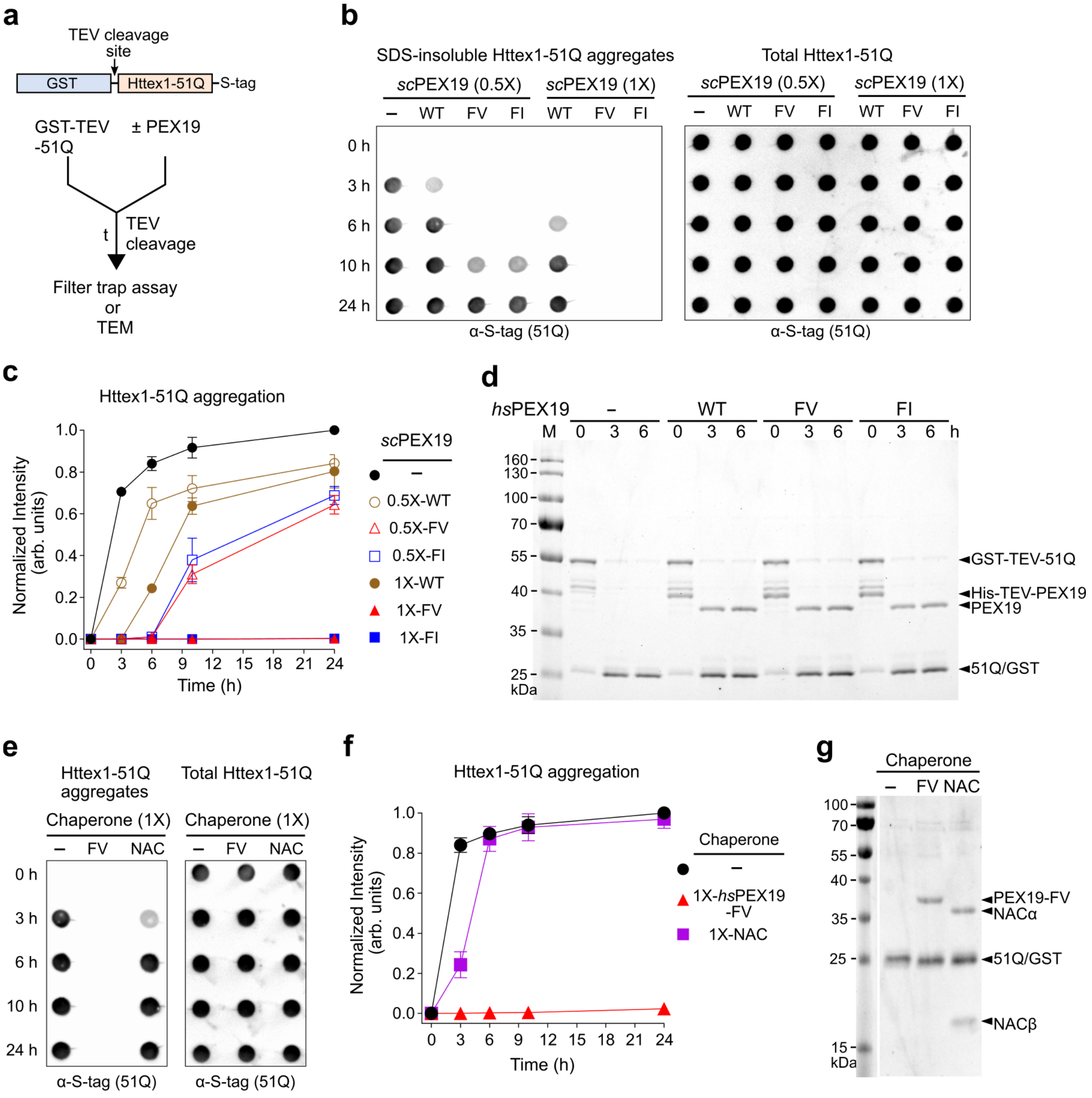

Supplementary Fig. 4. *hs*PEX19-FV is more effective in suppressing Httex1-51Q aggregation than NAC.

a, Schematic showing the *in vitro* aggregation assays used to measure SDS-insoluble Httex1-51Q aggregates or Httex1-51Q fibrils. TEV protease was added to GST-TEV-Httex1-51Q in the absence and presence of PEX19 (t=0). Upon adding TEV protease, the N-terminal GST tag of GST-TEV-Httex1-51Q was removed, and Httex1-51Q aggregation was initiated. The reactions were carried out in the absence and presence of PEX19-WT and its variants. At the indicated time, samples were taken and further processed for either filter trap assays or TEM measurements. The C-terminal S-tag enabled the detection of Httex1-51Q proteins in the filter trap assay.

b, **c**, *In vitro* aggregation assay of Httex1-51Q in the absence and presence of *sc*PEX19 proteins. 3 μM of GST-TEV-Httex1-51Q-Stag and 1.5 or 3 μM of *sc*PEX19 proteins were incubated at 30°C. After the addition of TEV protease, samples were quenched at the indicated time points. SDS-insoluble Httex1-51Q aggregates were detected on the cellulose acetate membrane, while total Httex1-51Q proteins were detected on the nitrocellulose membrane. SDS-insoluble Httex1-51Q aggregates in (**b**) and their replicates were quantified and are shown in (**c**) (n=3, mean ± SD).

d, SDS-PAGE analysis to monitor the TEV cleavage efficiency of Httex1-51Q. The same samples collected from **Fig. 2c** were used for SDS-PAGE analysis. The *hs*PEX19 proteins also contain the N-terminal His-tag with TEV protease cleavage site.

e, **f**, *In vitro* aggregation assay of Httex1-51Q in the presence of *hs*PEX19-FV or NAC proteins. 3 μM of GST-TEV-Httex1-51Q-Stag and 3 μM of *hs*PEX19-FV or NAC were incubated at 30°C and the samples at the indicated time were collected and processed for the filter trap assay. SDS-insoluble Httex1-51Q aggregates in (**e**) and their replicates were quantified and shown in (**f**) (n=3, mean ± SD).

g, SDS-PAGE analysis to monitor the TEV cleavage efficiency of Httex1-51Q. Samples from the 24 h timepoint collected for (**e**) were used for SDS-PAGE analysis. The difference in chaperone activities of *hs*PEX19-FV and NAC is likely not due to different TEV cleavage efficiency.

Source data are provided as a Source Data file.

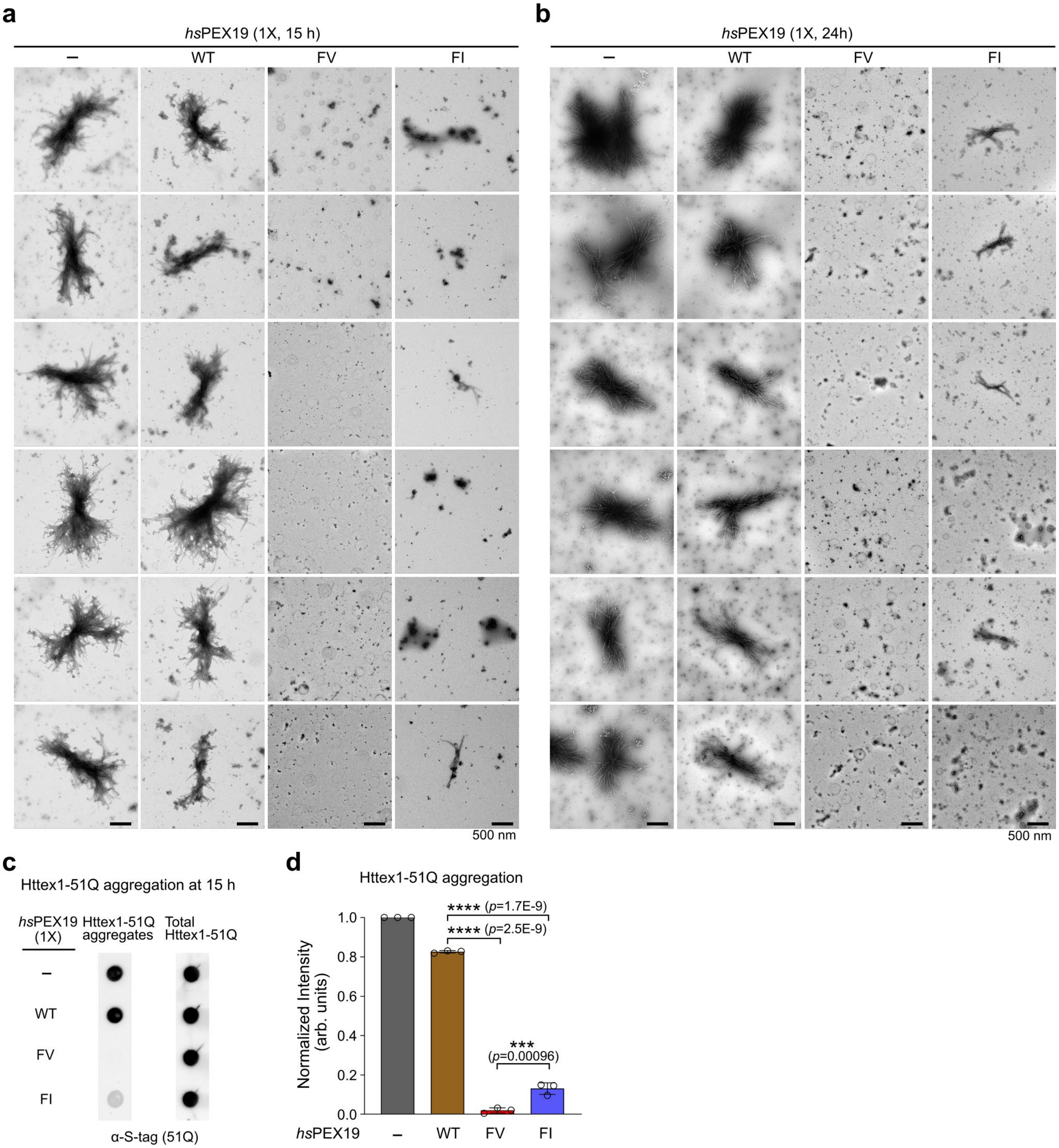

Supplementary Fig. 5. *hs*PEX19-FV inhibits fibril formation and SDS-insoluble aggregation of Httex1-51Q more effectively than *hs*PEX19-FI

a, **b**, Multiple representative TEM images of Httex1-51Q in the absence and presence of *hs*PEX19 proteins. Equimolar concentrations of GST-TEV-Httex1-51Q and *hs*PEX19 proteins were incubated for 15 h (**a**) and 24 h (**b**) at 30°C, and the samples were used for TEM analysis. Scale bar: 500 nm.

c, **d**, *In vitro* aggregation assay of Httex1-51Q in the absence and presence of *hs*PEX19 proteins. 3 μM of GST-TEV-Httex1-51Q and 3 μM of PEX19 proteins were incubated in the presence of TEV protease at 30°C for 15 h. After quenching the reactions, samples were analyzed using the filter trap assay. SDS-insoluble Httex1-51Q aggregates in (**c**) and their replicates were quantified, and the data are represented as mean ± SD (n = 3) in (**d**). Pairwise comparisons are shown as indicated, where ***$p < 0.001$, ****$p < 0.0001$ by the ordinary one-way ANOVA with Tukey post-hoc test.

Source data are provided as a Source Data file.

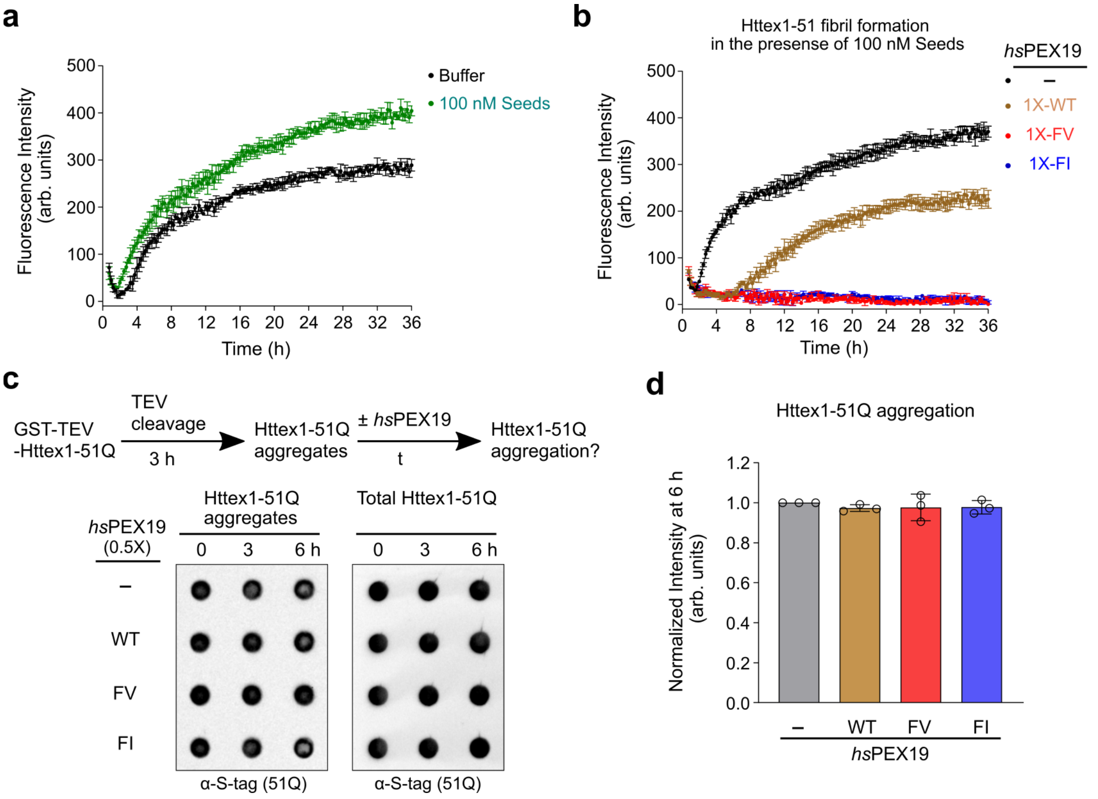

Supplementary Fig. 6. *hs*PEX19 variants act as a holdase that prevents the initial aggregation of Httex1-51Q.

a, b, ThioflavinT fluorescence assay to measure fibril formation of Httex1-51Q in the presence of 100 nM seeds. 3 μM of Httex1-51Q was incubated for 24 h at 30°C, and the sample was probe-sonicated to generate seeds. To monitor seed-promoted fibril formation of Httex1-51Q, ~50% of the TEV concentration used in **Fig. 2e** was supplemented with 3 μM of Httex1-51Q (**a**) or both Httex1-51Q and *hs*PEX19 proteins (**b**). Both samples in (**a**) and (**b**) were loaded onto the same 96-well plate, and the fluorescence intensities were measured every 15 min. Data are represented as mean ± SD with n = 3.

c, d, *In vitro* aggregation assay to monitor the disaggregase activity of *hs*PEX19 proteins. 3 μM of Httex1-51Q was preincubated with TEV protease at 30°C. After 3 h incubation with TEV protease, either *hs*PEX19-WT or its variants were added to a final concentration of 1.5 μM and further incubated for 3 and 6 h. Samples were then subjected to the filter trap assay. SDS-insoluble Httex1-51Q aggregates in (**c**) and their replicates were quantified, and the data are represented as mean ± SD (n = 3) in (**d**).

Source data are provided as a Source Data file.

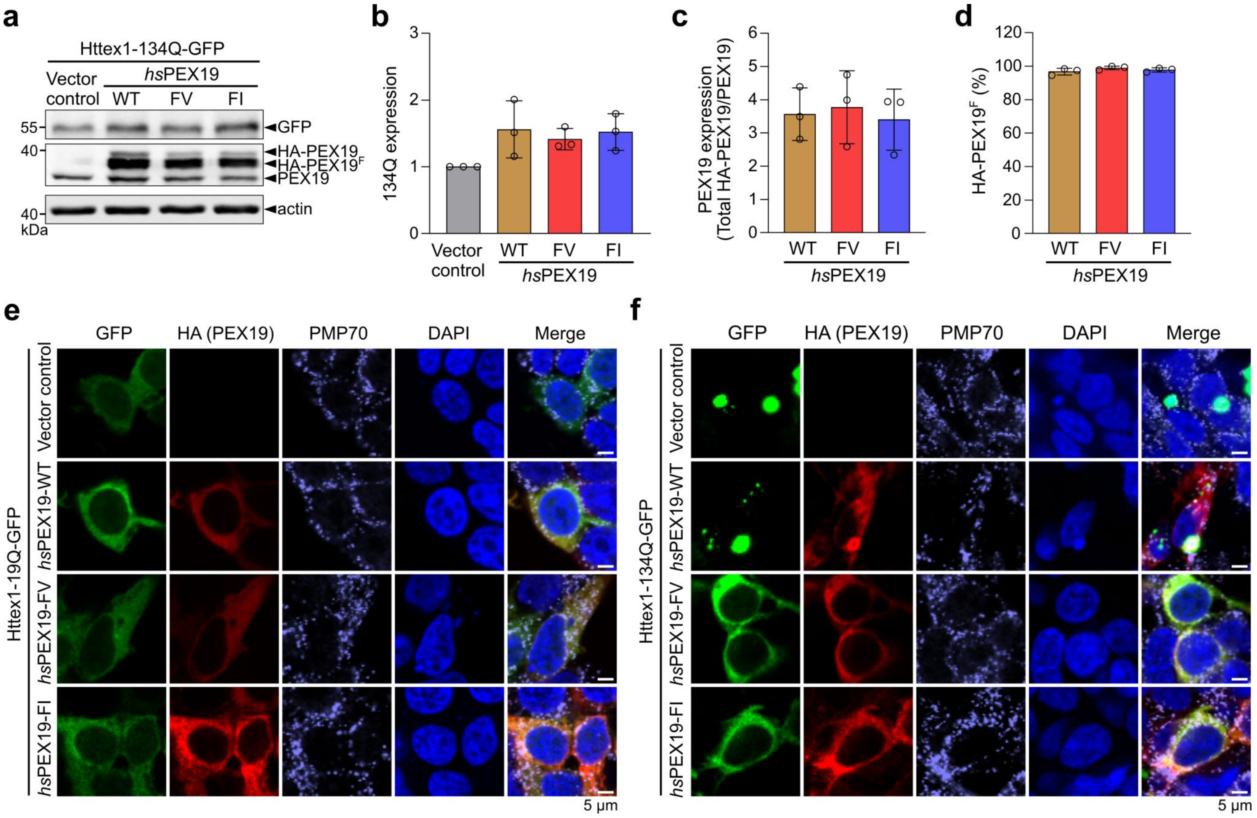

Supplementary Fig. 7. *hs*PEX19 variants reduce the cytosolic aggregation of Httex1-134Q in HEK293T cells.

a-**d**, (**a**) Representative western blot images showing protein levels of Httex1-134Q-GFP and *hs*PEX19 coexpressed in HEK293T cells. (**b**-**d**), Quantification of the data in (**a**). The expression levels of both Httex1-134Q-GFP in (**b**) and *hs*PEX19 in (**c**) were normalized relative to β-actin. (**d**) Relative expression levels of the farnesylated HA-PEX19 (denoted HA-PEX19^F) were calculated as (Intensity of HA-PEX19^F band/Total intensity of HA-PEX19 and HA-PEX19^F bands)*100. Data in (**b**-**d**) are shown as mean ± SD, with three biological replicates (n=3).

e, **f**, Confocal microscopy images of HEK293T cells coexpressing Httex1-19Q-GFP or Httex1-134Q-GFP and *hs*PEX19. HA-*hs*PEX19 and PMP70 were stained using HA and PMP70 antibodies, respectively. Representative images from three biological replicates are shown (n=3).

Source data are provided as a Source Data file.

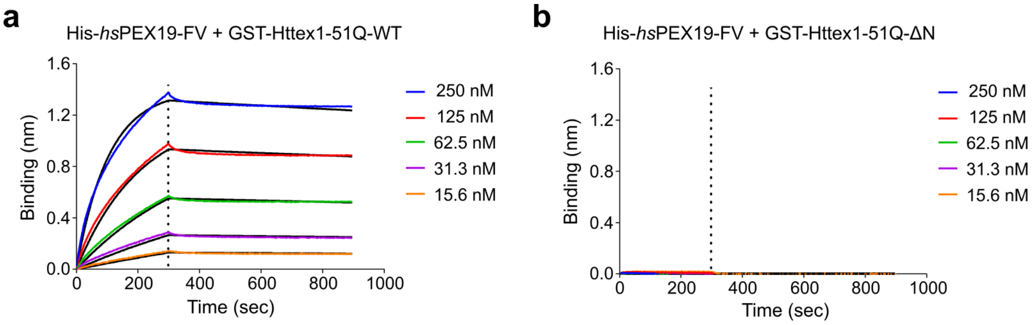

Supplementary Fig. 8. The N17 domain of Httex1-51Q is a primary binding domain for *hs*PEX19-FV.

a, **b** Representative biolayer interferometry (BLI) sensograms of GST-Httex1-51Q-WT (**a**) or GST-Httex11-51Q-ΔN (**b**) binding to immobilized His-*hs*PEX19-FV. Fitted curves are shown as black lines. Kinetic analysis in (**a**) showed that GST-Httex1-51Q-WT readily binds to *hs*PEX19-FV (an estimated dissociation constant (K_D) of 2.5 ± 0.26 nM, association rate constant (k_{on}) of $4.1 \times 10^4 \pm 5.86 \times 10^3$ $M^{-1}s^{-1}$, and dissociation rate constant (k_{off}) of $1 \times 10^{-4} \pm 3.64 \times 10^{-6}$ s^{-1}). The data are shown as mean ± SD (n=3). Due to incomplete curves in the association and dissociation steps, these estimated values may be different from the actual kinetic values.

Source data are provided as a Source Data file.

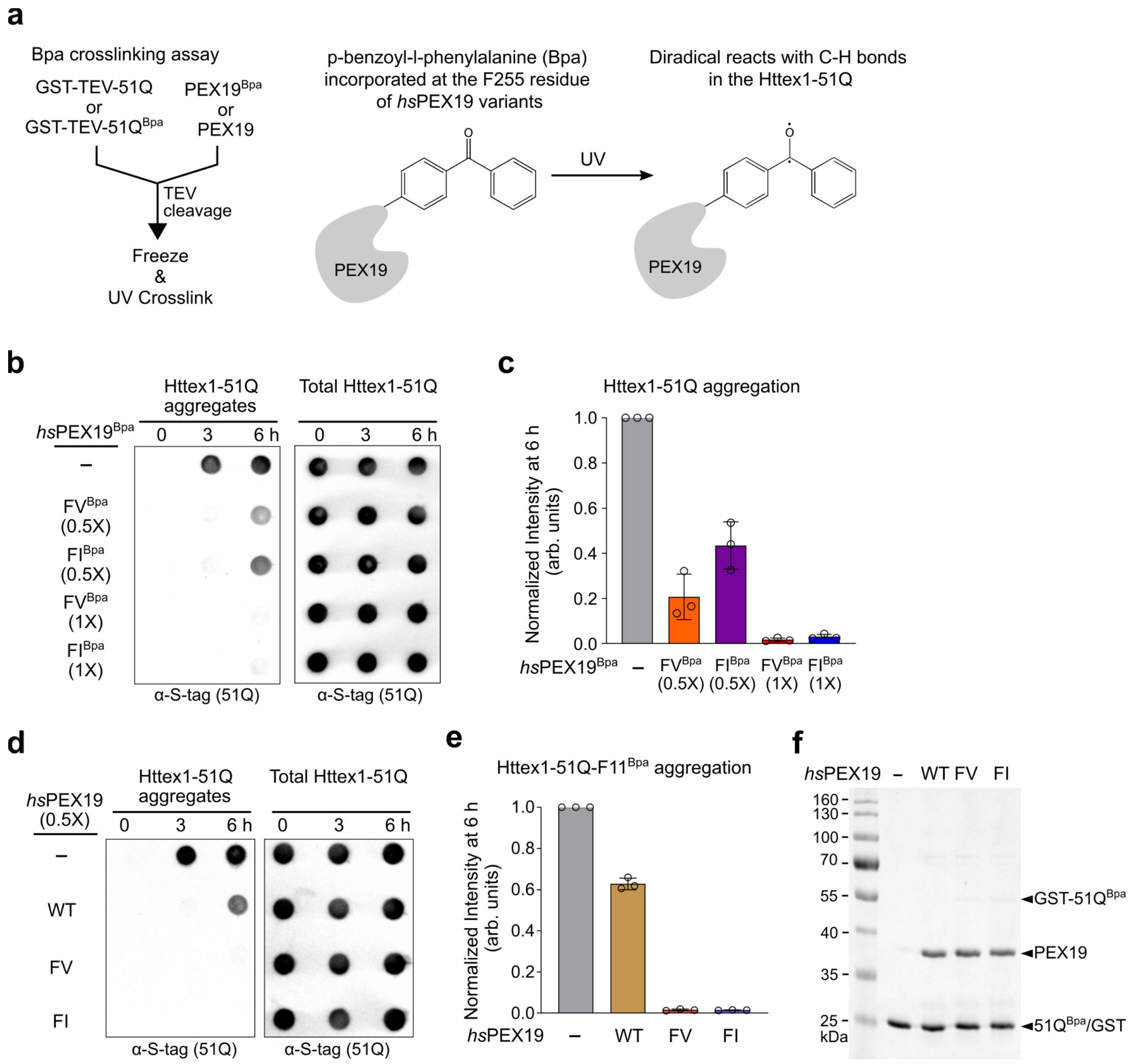

Supplementary Fig. 9. Bpa crosslinking assay to monitor the interaction of Httex1-51Q and *hs*PEX19 variant.

a, Schematic representation of Bpa crosslinking assay. GST-TEV-Httex1-51Q and *hs*PEX19^{Bpa} or GST-TEV-Httex1-51^{Bpa} and *hs*PEX19 were incubated in the presence of TEV protease at 30 °C. After 3 h, the samples were frozen and analyzed by UV crosslinking at -20 °C. Under UV, the diradical species at the F255^{Bpa} residue of *hs*PEX19 variants react with C-H bonds in Httex1-51Q within an estimated radius of ~3.1 Å from the oxygen atom[1].

b, **c**, *In vitro* aggregation assay to monitor the chaperone activity of *hs*PEX19-FV^{Bpa} and *hs*PEX19-FI^{Bpa} toward Httex1-51Q-WT. 3 μM of Httex1-51Q-WT was incubated with 1.5 or 3 μM of *hs*PEX19-FV^{Bpa} or *hs*PEX19-FI^{Bpa} at 30°C. Since 3 μM of both *hs*PEX19-FV^{Bpa} and *hs*PEX19-FI^{Bpa} were sufficient to prevent the aggregation of Httex1-51Q-WT, we carried out the Bpa crosslinking assay with the same reaction conditions in **Fig. 3h** and **3i**. SDS-insoluble

Httex1-51Q aggregates in (**b**) and their replicates were quantified, and the data are represented as mean ± SD (n = 3) in (**c**).

d-f, *In vitro* aggregation assay of Httex1-51Q-F11^{Bpa} was carried out with 1.5 μM of *hs*PEX19 protein. SDS-insoluble Httex1-51Q aggregates in (**d**) and their replicates were quantified, and the data are represented as mean ± SD (n = 3) in (**e**). The same reaction samples at 3 h were applied for the SDS-PAGE analysis in (**f**).

Source data are provided as a Source Data file.

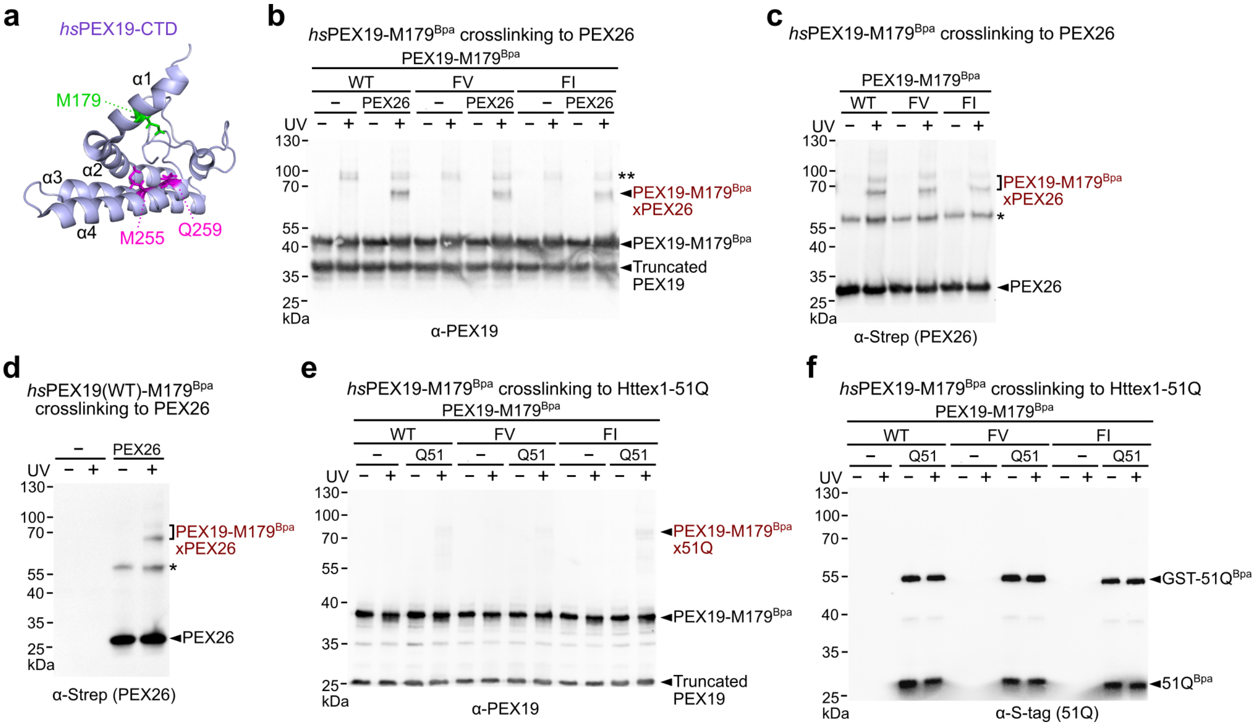

Supplementary Fig. 10. Httex1-51Q does not interact with the α1 helix of *hs*PEX19 proteins

a, The NMR structure of PEX19-CTD (PDB 5LNF)[2]. The M179 residue located in the α1 helix of PEX19 is known to interact with PEX26[3].

b-**d**, Bpa crosslinking assay to monitor the interaction of *hs*PEX19-M179^{Bpa} and PEX26. Bpa was incorporated at the M179 residue in *hs*PEX19-WT and its variants. Crosslinked samples were analyzed using Western blots probed with PEX19 (**b**) and Strep (51Q) (**c** and **d**) antibodies. ** represents a mixture of PEX19-M179^{Bpa}xPEX19 and PEX19-M179^{Bpa}xPEX26 bands in (**b**). For better separation between PEX19-M179^{Bpa}xPEX26 from the mixture of crosslinked products, the reaction was run on 6% Tricine gels. "*" represents the SDS-resistant PEX26 dimers in (**c**) and (**d**).

e, **f**, Bpa-crosslinking assay to monitor the interaction of *hs*PEX19-M179^{Bpa} and Httex1-51Q. Crosslinked samples ran onto 10% Glycine gels and were analyzed using Western blots probed with PEX19 (**e**) and S-tag (51Q) (**f**) antibodies.

Data in (**b**, **c**, **e**, **f**) and (**d**) were collected from three and two independent experiments, respectively.

Source data are provided as a Source Data file.

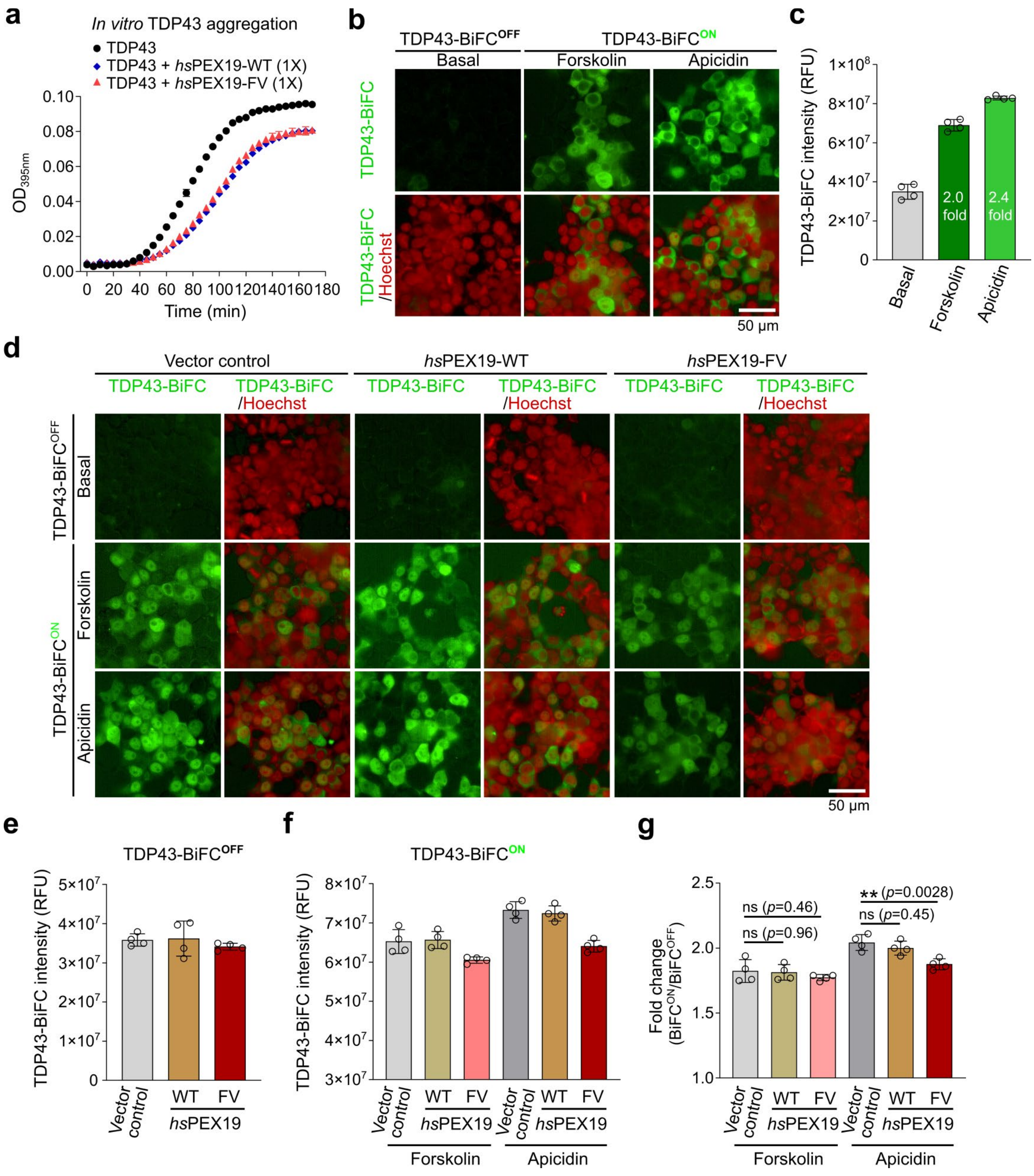

Supplementary Fig. 11. *hs*PEX19 variants do not efficiently prevent TDP43 aggregation *in vitro* and in mammalian cells.

a, *In vitro* TDP43 aggregation assay. TDP43 aggregation was initiated by adding TEV protease in the reactions containing TDP43-TEV-MBP-His$_6$ and *hs*PEX19 proteins, and the turbidity caused by TDP43 aggregation was monitored at the optical density of 395 nm as described in Methods. Data in (**a**) are shown as mean ± SEM, with n=2. Error bars are shown but may not be visible in some cases.

b, **c**, Activation of TDP-43 oligomerization in HEK293 TDP43-BiFC cells. Representative microscopic images of Forskolin- and Apicidin-activated TDP43-BiFC cells are shown in (**b**). The fluorescence intensities of TDP43-BiFC in (**b**) and its replicates were quantified in (**c**). Intensity fold changes of Forskolin- and Apicidin-activated TDP43-BiFC are approximately 2.0 and 2.4, respectively.

d-g, The *hs*PEX19-transfected TDP43-BiFC cells were treated with Forskolin and Apicidin, and representative microscopy images are shown in (**d**). The fluorescence intensities of TDP43-BiFC^{OFF} and reagent-activated TDP43-BiFC^{ON} are quantified in (**e**) and (**f**), respectively. The intensity fold changes are also summarized in (**g**).

All data in (**c**), (**e**), (**f**), and (**g**) are shown as mean ± SD, with n=4 (biological replicates). Pairwise comparisons are shown as indicated, where $**p < 0.01$ by the ordinary one-way ANOVA with Tukey post-hoc test.

Source data are provided as a Source Data file.

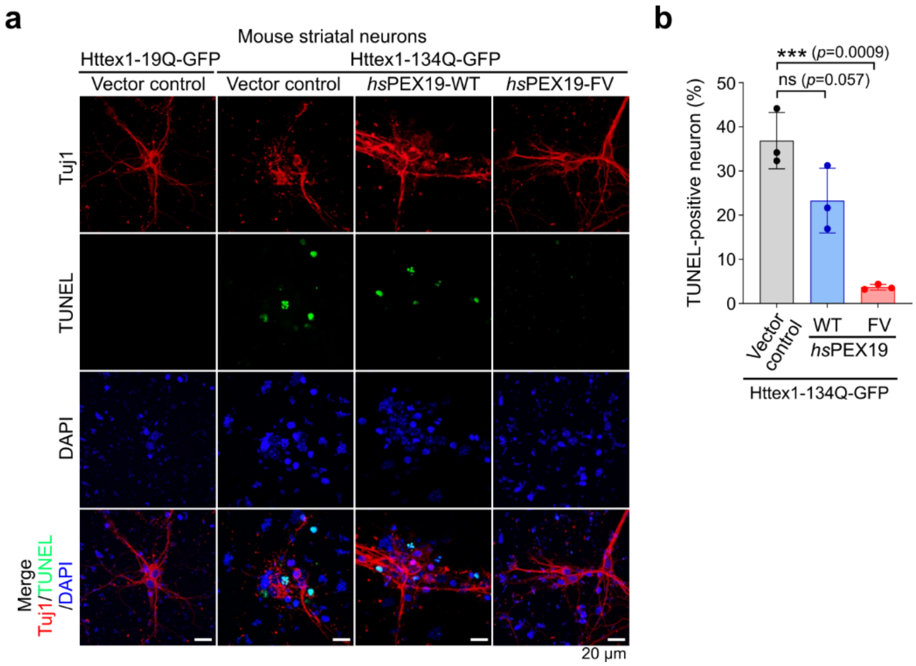

Supplementary Fig. 12. Overexpression of *hs*PEX19-FV rescued mHttex1-associated neurotoxicity in striatal neurons.

a, **b**, TUNEL cell death assay in mouse primary striatal neurons coexpressing Httex1-19Q-GFP and Httex1-134Q-GFP with vector control, *hs*PEX19-WT, or *hs*PEX19-FV. (**a**) Representative confocal microscopy images labeled with anti-Tuj1 (red), TUNEL (green), and DAPI (blue). (**b**) Quantification of TUNEL-positive neurons in (**a**) and their replicates. The total numbers of neurons for Vector control, *hs*PEX19-WT, and *hs*PEX19-FV are 382, 371, and 224, respectively. The data are represented as mean ± SD (n = 3, biological replicates). Pairwise comparisons are shown as indicated, where ***p < 0.001 by the ordinary one-way ANOVA with Tukey post-hoc test.

Source data are provided as a Source Data file.

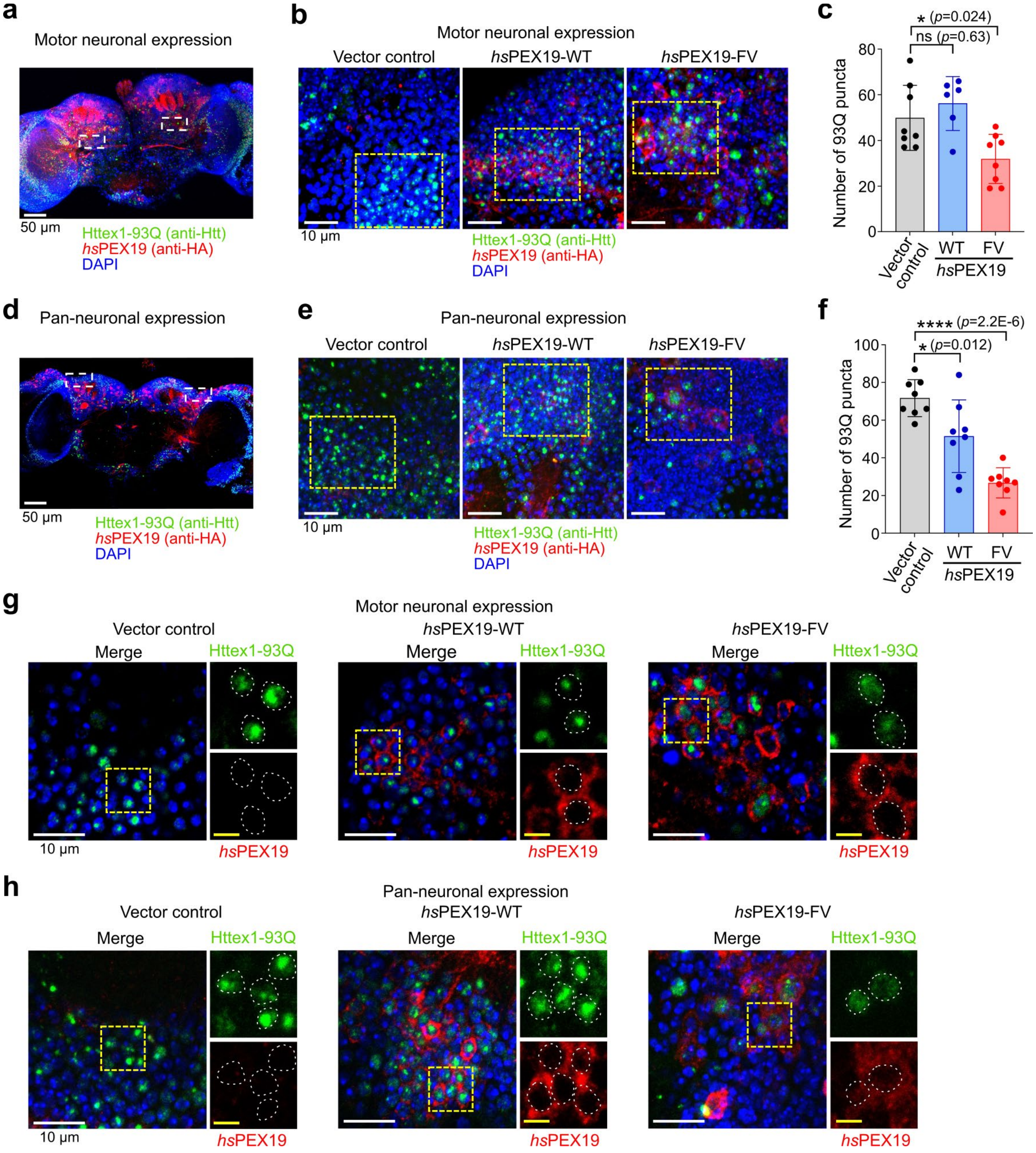

Supplementary Fig. 13. Overexpression of *hs*PEX19-FV reduced nuclear puncta formation of Httex1-93Q in both motor- and pan-neurons.

a, Representative whole merged image of fly adult brain coexpressing Httex1-93Q and *hs*PEX19-WT in motor neurons at day 12 after pupal eclosion (APE). The N-terminally HA-tagged *hs*PEX19 and Httex1-93Q proteins were stained using HA and Htt (mEM48) antibodies, respectively. The white dashed squares represent the regions used for the analysis of the Httex1-93Q puncta number. Scale bar: 50 μm.

b, **c**, (**b**) Representative images of motor neurons coexpressing Httex1-93Q and vector control,

*hs*PEX19-WT or *hs*PEX19-FV at day 12 APE. The same-sized of areas (yellow dashed squares) was used to quantify the number of Httex1-93Q puncta in (**c**). Scale bar: 10 µm. (**c**) Quantification of Httex1-93Q puncta number. The data in (**c**) are shown as mean ± SEM, with n = 6-8 motor neuron regions from 3 or 4 adult fly brains for each condition (Vector control, n=8; *hs*PEX19-WT, n=6; *hs*PEX19-FV, n=8). Statistical significance was evaluated using the ordinary one-way ANOVA with Tukey post-hoc test. **p*<0.05, ns=not significant.

d, Representative whole merged image of fly adult brain coexpressing Httex1-93Q and *hs*PEX19-WT in pan-neurons at day 10 APE. The white dashed squares represent the regions used for the analysis of the Httex1-93Q puncta number within Kenyon cell somata. Scale bar: 50 µm.

e, **f**, (**e**) Representative images of pan-neurons coexpressing Httex1-93Q and vector control, *hs*PEX19-WT or *hs*PEX19-FV at day 10 APE. (**f**) Quantification of Httex1-93Q puncta number. The data in (**f**) are shown as mean ± SEM, with 8 kenyon cell somata (n = 8) from 4 adult fly brains for each condition. Statistical significance was evaluated using the ordinary one-way ANOVA with Tukey post-hoc test. *p<0.05, ****p*<0.0001.

g, **h**, Representative images of expressing Httex1-93Q and vector control, *hs*PEX19-WT or *hs*PEX19-FV in motor neurons (**g**) at day 12 APE or pan-neurons (**h**) at day 10 APE. Scale bar (white solid line), 10 µm; Inset images (yellow dashed squares) are 2-fold enlarged, and the white dashed lines indicate the nucleus. Scale bar (yellow solid line): 5 µm.

Source data are provided as a Source Data file.

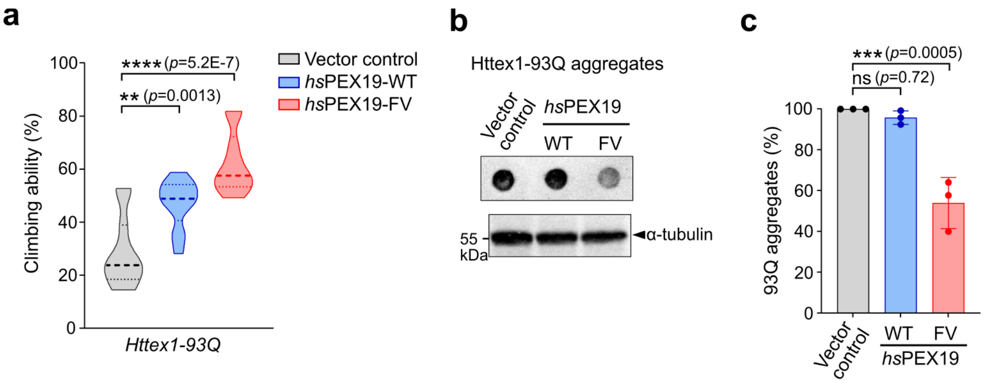

Supplementary Fig. 14. Fly adult brains coexpressing Httex1-93Q and *hs*PEX19-FV exhibit lower levels of Httex1-93 aggregates.

a, Climbing ability of 15-day-old adult flies (*Httex1-93Q*) expressing vector control, *hs*PEX19-WT, and *hs*PEX19-FV in pan-neurons. Climbing index (5 cm/6 sec). The data in (**a**) are shown as violin plots with mean and quartiles (from left to right, n=137, 157, 133 adult flies). Statistical significance was evaluated using the one-way ANOVA with Tukey post-hoc test. $^{**}p<0.01$, $^{****}p<0.0001$, ns=not significant.

b,**c**, **(b)** (Top) A representative filter trap assay image to determine chaperone activity of *hs*PEX19-FV in *Httexl-93Q*-expressing 15-day-old adult flies. (Bottom) A western blot image of α-tubulin shows consistent protein loading across the samples in the filter trap assay. SDS-insoluble Httex1-93Q aggregates in (**b**) and their replicates were quantified. The data are represented as mean ± SD (n=3, biological replicates) in (**c**). Statistical significance was evaluated using the one-way ANOVA with Tukey post-hoc test. $^{***}p<0.001$, ns=not significant.

Source data are provided as a Source Data file.

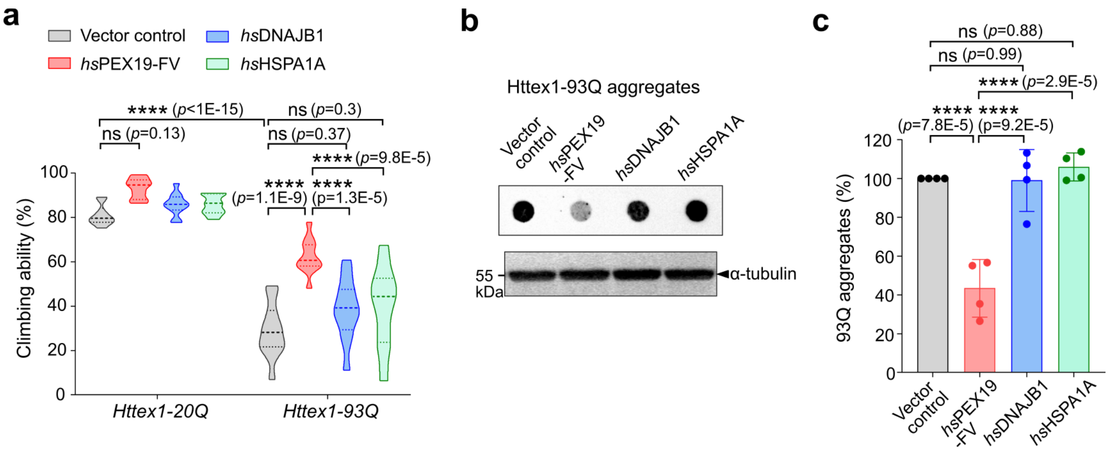

Supplementary Fig. 15. *hs*PEX19-FV rescued the Httex1-93Q aggregation-associated neurotoxicity more effectively than *hs*DNAJB1.

a, Climbing ability of 15-day-old adult flies (*Httex1-20Q* and *Httex1-93Q*) expressing vector control, *hs*PEX19-FV, *hs*DNAJB1, and *hs*HSPA1A in pan-neurons. Climbing index (5 cm/6 sec). The data are shown as violin plots with mean and quartiles (from left to right n= 130, 153, 157, 115, 139, 185, 225, 105 adult flies). Statistical significance was evaluated using the two-way ANOVA with Tukey post-hoc test. ****p<0.0001, ns=not significant.

b, c, (**b**) (Top) A representative filter trap assay image to compare chaperone activities of *hs*PEX19-FV with *hs*DNAJB1 or *hs*HSPA1A in *Httexl-93Q*-expressing 15-day-old adult flies. (Bottom) α-tubulin in the western blot image serves as a loading control for the filter trap assay. (**c**) SDS-insoluble Httex1-93Q aggregates in (**b**) and their replicates were quantified. The data are represented as mean ± SD (n= 4, biological replicates) in (**c**). Statistical significance was evaluated using the one-way ANOVA with Tukey post-hoc test. ****p<0.0001, ns=not significant.

Source data are provided as a Source Data file.

Supplementary Table 1. Genotypes of fly lines.

The genotypes of transgenic flies used in this study are listed. *Elav-Gal4* and *D42-Gal4* drivers were used to express transgenes in pan-neurons and motor neurons, respectively.

Drivers	Fly lines	Genotypes
Elav-GAL4	W^{1118}/vector control	*Elav-GAL4/+;UAS-pACU2 vector/+*
	W^{1118}/hsPEX19-WT	*Elav-GAL4/+;UAS-HA-hsPEX19-WT/+*
	W^{1118}/hsPEX19-FV	*Elav-GAL4/+;UAS-HA-hsPEX19-FV/+*
	Httex1-20Q/vector control	*Elav-GAL4/+;UAS-pACU2 vector/UAS-Httex1-20Q*
	Httex1-20Q/ hsPEX19-WT	*Elav-GAL4/+;UAS-HA-hsPEX19-WT/UAS-Httex1-20Q*
	Httex1-20Q/ hsPEX19-FV	*Elav-GAL4/+;UAS-HA-hsPEX19-FV/UAS-Httex1-20Q*
	Httex1-20Q/ hsDNAJB1	*Elav-GAL4/+;UAS-HA-hsDNAJB1/UAS-Httex1-20Q*
	Httex1-20Q/ hsHSPA1A	*Elav-GAL4/UAS-HA-hsHSPA1A;UAS-Httex1-20Q/+*
	Httex1-93Q/vector control	*Elav-GAL4/+;UAS-pACU2 vector/UAS-Httex1-93Q*
	Httex1-93Q/ hsPEX19-WT	*Elav-GAL4/+;UAS-HA-hsPEX19-WT/UAS-Httex1-93Q*
	Httex1-93Q/ hsPEX19-FV	*Elav-GAL4/+;UAS-HA-hsPEX19-FV/UAS-Httex1-93Q*
	Httex1-93Q/ hsDNAJB1	*Elav-GAL4/+;UAS-HA-hsDNAJB1/UAS-Httex1-93Q*
	Httex1-93Q/ hsHSPA1A	*Elav-GAL4/UAS-HA-hsHSPA1A;UAS-Httex1-93Q/+*
D42-GAL4	W^{1118}/vector control	*UAS-pACU2 vector/+;D42-GAL4/+*
	W^{1118}/hsPEX19-WT	*UAS-HA-hsPEX19-WT/+;D42-GAL4/+*
	W^{1118}/hsPEX19-FV	*UAS-HA-hsPEX19-FV/+;D42-GAL4/+*
	Httex1-20Q/vector control	*UAS-pACU2 vector/+;D42-GAL4/UAS-Httex1-20Q*
	Httex1-20Q/ hsPEX19-WT	*UAS-HA-hsPEX19-WT/+;D42-GAL4/UAS-Httex1-20Q*
	Httex1-20Q/ hsPEX19-FV	*UAS-HA-hsPEX19-FV/+;D42-GAL4/UAS-Httex1-20Q*
	Httex1-93Q/vector control	*UAS-pACU2 vector/+;D42-GAL4/UAS-Httex1-93Q*
	Httex1-93Q/ hsPEX19-WT	*UAS-HA-hsPEX19-WT/+;D42-GAL4/UAS-Httex1-93Q*
	Httex1-20Q/ hsPEX19-FV	*UAS-HA-hsPEX19-FV/+;D42-GAL4/UAS-Httex1-93Q*

References

1. Dormán, G. & Prestwich, G. D. Benzophenone photophores in biochemistry. *Biochemistry* **33**, 5661–5673 (1994).
2. Emmanouilidis, L. *et al.* Allosteric modulation of peroxisomal membrane protein recognition by farnesylation of the peroxisomal import receptor PEX19. *Nat Commun* **8**, 14635 (2017).
3. Oh, J., Kim, D. K., Ahn, S. H., Kim, H. M. & Cho, H. A dual role of the conserved PEX19 helix in safeguarding peroxisomal membrane proteins. *iScience* **27**, (2024).
