# Supplementary material for: Engineering a membrane protein chaperone to ameliorate the proteotoxicity of mutant huntingtin
Source: Nat Commun. 2025 Jan 17;16:737. doi: 10.1038/s41467-025-56030-6 (PMC11742450; doi:10.1038/s41467-025-56030-6)
Supplement: Supplementary file 2 — Reporting Summary [file 41467_2025_56030_MOESM2_ESM.pdf]

nature portfolio

Corresponding author(s): Ho Min Kim
Sung Bae Lee
Hyunju Cho

Last updated by author(s): Dec 19, 2024

Reporting Summary

Nature Portfolio wishes to improve the reproducibility of the work that we publish. This form provides structure for consistency and transparency in reporting. For further information on Nature Portfolio policies, see our Editorial Policies and the Editorial Policy Checklist.

Statistics

For all statistical analyses, confirm that the following items are present in the figure legend, table legend, main text, or Methods section.

n/a	Confirmed	
☐	☒	The exact sample size (*n*) for each experimental group/condition, given as a discrete number and unit of measurement
☐	☒	A statement on whether measurements were taken from distinct samples or whether the same sample was measured repeatedly
☐	☒	The statistical test(s) used AND whether they are one- or two-sided<br>*Only common tests should be described solely by name; describe more complex techniques in the Methods section.*
☒	☐	A description of all covariates tested
☐	☒	A description of any assumptions or corrections, such as tests of normality and adjustment for multiple comparisons
☐	☒	A full description of the statistical parameters including central tendency (e.g. means) or other basic estimates (e.g. regression coefficient) AND variation (e.g. standard deviation) or associated estimates of uncertainty (e.g. confidence intervals)
☐	☒	For null hypothesis testing, the test statistic (e.g. *F*, *t*, *r*) with confidence intervals, effect sizes, degrees of freedom and *P* value noted<br>*Give P values as exact values whenever suitable.*
☒	☐	For Bayesian analysis, information on the choice of priors and Markov chain Monte Carlo settings
☒	☐	For hierarchical and complex designs, identification of the appropriate level for tests and full reporting of outcomes
☒	☐	Estimates of effect sizes (e.g. Cohen's *d*, Pearson's *r*), indicating how they were calculated

Our web collection on statistics for biologists contains articles on many of the points above.

Software and code

Policy information about availability of computer code

Data collection	iBrightTM FL1000 imaging system; BioTek Epoch 2 plate reader; FEI TecnaiTM G2 spirit microscope; Eclipse Ti-E microscope; Zeiss LSM 700 confocal microscope; Operetta CLS High-Content Analysis System; Infinite M Plex microplate; Octet R8 Protein Analysis System
Data analysis	iBirghtTM Analysis software v5.4.0; GraphPad Prism v10; Gen 5 v2.09 software ; Harmony v4.9 software; NIS-Elements software (Nikon) v 5.21.00; Fiji software Java 1.8.0_172; Octet Analysis Studio 12 software v13

For manuscripts utilizing custom algorithms or software that are central to the research but not yet described in published literature, software must be made available to editors and reviewers. We strongly encourage code deposition in a community repository (e.g. GitHub). See the Nature Portfolio guidelines for submitting code & software for further information.

Data

Policy information about availability of data

All manuscripts must include a data availability statement. This statement should provide the following information, where applicable:

- Accession codes, unique identifiers, or web links for publicly available datasets
- A description of any restrictions on data availability
- For clinical datasets or third party data, please ensure that the statement adheres to our policy

All data generated in this study are provided in the manuscript's main text (Figures 1-5), Supplementary information (Supplementary Figures 1-15), and Source Data file. A previously published structure of PEX19 used to show both mutation sites and Bpa incorporation positions is available under PDB code 5LNF [https://doi.org/10.2210/pdb5LNF/pdb]. Source data are provided in this paper.

Research involving human participants, their data, or biological material

Policy information about studies with human participants or human data. See also policy information about sex, gender (identity/presentation), and sexual orientation and race, ethnicity and racism.

Reporting on sex and gender	n/a
Reporting on race, ethnicity, or other socially relevant groupings	n/a
Population characteristics	n/a
Recruitment	n/a
Ethics oversight	n/a

Note that full information on the approval of the study protocol must also be provided in the manuscript.

Field-specific reporting

Please select the one below that is the best fit for your research. If you are not sure, read the appropriate sections before making your selection.

☒ Life sciences ☐ Behavioural & social sciences ☐ Ecological, evolutionary & environmental sciences

For a reference copy of the document with all sections, see nature.com/documents/nr-reporting-summary-flat.pdf

Life sciences study design

All studies must disclose on these points even when the disclosure is negative.

Sample size	No sample size calculation was performed. The sample size (n) of each experiment is indicated in the figure legends in the main manuscript and Supplementary information file. All Drosophila climbing assays and lifespan analyses were performed with sample numbers (n≥91) (Sample size was chosen based upon the previous studies in the field, ref: PMID 35927252 and PMID 37291115).
Data exclusions	No data were excluded from the data analyses.
Replication	The number of replicates are specified in the figure legends. Biochemical measurements were independently repeated 2-3 times. Cellular experiments have at least three biological replicates.
Randomization	Flies of the same genotype were randomly selected for each experiment.
Blinding	Investigators were not blinded, as for each experiment the same investigator performed crosses, data collection and analysis.

Reporting for specific materials, systems and methods

We require information from authors about some types of materials, experimental systems and methods used in many studies. Here, indicate whether each material, system or method listed is relevant to your study. If you are not sure if a list item applies to your research, read the appropriate section before selecting a response.

Materials & experimental systems

n/a	Involved in the study	
	x	Antibodies
	x	Eukaryotic cell lines
x		Palaeontology and archaeology
	x	Animals and other organisms
x		Clinical data
x		Dual use research of concern
x		Plants

Methods

n/a	Involved in the study	
x		ChIP-seq
x		Flow cytometry
x		MRI-based neuroimaging

Antibodies

Antibodies used

THE™ NWSHPQFEK (Strep) Tag antibody (1:3000, Genscripts , Cat# A01732)
THE™ His Tag antibody (1:3000, Genscripts, Cat# A00186)
THE™ DYKDDDDK Tag antibody (1:3000, Genscripts, Cat# A00187)
GFP antibody for Western Blot (1:3000, Sigma-Aldrich, Cat# SAB4301138)
PGK1 antibody [22C5D8] (1:3000, Abcam, Cat# ab113687)
PEX19 antibody (533) (1:3000, Novus Biologicals, Cat# NBP2-43757)
HA Tag antibody (2-2.2.14) for Immunofluorescence (1:100, Invitrogen, Cat# 26183)
HA Tag antibody (C29F4) for Immunohistochemistry (1:200, Cell Signaling, Cat# 3724S)
Huntingtin antibody (mEM48) (1:200, Sigma-Aldrich, Cat# MAB5374)
beta Actin antibody (8H10D10) (1:5000, Invitrogen, Cat# MA5-15452)
IRDye® 800CW Goat anti-Mouse IgG Secondary antibody (1:15000, Li-Cor, Cat# 926-32210)
IRDye® 800CW Goat anti-Rabbit IgG Secondary antibody (1:15000, Li-Cor, Cat# 926-32211)
Goat anti-Mouse IgG (H+L) Secondary antibody, Alexa Fluor™ 488 (1:200, Invitrogen, Cat# A-11001)
Goat anti-Rabbit IgG (H+L) Secondary antibody, Alexa Fluor™ 555 (1:200, Invitrogen, Cat# A-21428)
Goat anti-Mouse IgG (H+L) Secondary antibody, Alexa Fluor™ 647 (1:1000, Invitrogen, Cat# A-21235)
Goat anti-Rabbit IgG (H+L) Secondary antibody, Alexa Fluor™ 568 (1:1000, Invitrogen, Cat# A-11011)
Cy™3 AffiniPure™ F(ab')$_2$ Fragment Donkey Anti-Guinea Pig IgG (H+L) Secondary antibody (1:1000, Jackson ImmunoResearch, Cat# 706-166-148)
Tuj1 antibody (1:500, Cat# ab18207)
HTT (MW8) antibody (1:1000, DSHB, Cat# concentrate 0.1 mL)
S-peptide Epitope Tag Monoclonal Antibody (6.2) (1:3000, Invitrogen, Cat# MA1-981)
PMP70 Polyclonal Antibody (1:200, Invitrogen, Cat# PA1-650)

Validation

Validation of all primary and secondary antibodies can be found on the manufacturer's website.

Eukaryotic cell lines

Policy information about cell lines and Sex and Gender in Research

Cell line source(s)

HEK293T cell line was originally obtained from American Type Culture Collection (ATCC CRL-3216). Using HEK293 cell line (ATCC CRL-1573), HEK293 TDP43-BiFC cell line was generated in house (Korea Institute of Science and Technology, Seoul, South Korea).

Authentication

None of the cell lines used were authenticated.

Mycoplasma contamination

We confirmed that all cell lines were tested negative for Mycoplasma contamination.

Commonly misidentified lines (See ICLAC register)

No commonly misidentified cell lines were used.

Animals and other research organisms

Policy information about studies involving animals; ARRIVE guidelines recommended for reporting animal research, and Sex and Gender in Research

Laboratory animals

Mice were housed under a 12 h light/dark cycle (light cycle: 7 AM–7 PM) at 20–26 °C with 40–60% humidity. We used postnatal 1 (P1) pups of ICR mice (DBL Co., Korea).
Transgenic fly lines are obtained from the Bloomington Drosophila Stock Center: W1118 (BL5905), UAS-Httex1-20Q (BL68412), UAS-Httex1-93Q (BL68418), UAS-hsDNAJB1 (stock #82244), UAS-hsHSPA1A (stock #97467), Elav-Gal4 (BL8765), and D42-Gal4 (BL8816). Several fly lines are generated by BestGene, Inc : UAS-vector control, UAS-hsPEX19-WT, and UAS-hsPEX19-FV. They had reached the ages of 10, 12, and 15 days at 27 °C prior to each experiment.

Wild animals

This study did not involve wild animals.

Reporting on sex	Male flies are only used for all experiment. For mice, it would not be possible to determine sex of 1-day-old pups.
Field-collected samples	This study did not involve samples collected from the field.
Ethics oversight	All experiments involving animal procedures were approved by Institutional Animal Care and Use Committees (IACUC) of IBS (Deajeon, Korea). No ethical approval is required for experiments with Drosophila melanogaster.

Note that full information on the approval of the study protocol must also be provided in the manuscript.

Plants

Seed stocks	n/a
Novel plant genotypes	n/a
Authentication	n/a
